# The MentalPlus® Digital Game Might Be an Accessible Open Source Tool to Evaluate Cognitive Dysfunction in Heart Failure with Preserved Ejection Fraction in Hypertensive Patients: A Pilot Exploratory Study

**DOI:** 10.1155/2018/6028534

**Published:** 2018-08-06

**Authors:** Valeria Fontenelle Angelim Pereira, Livia Stocco Sanches Valentin

**Affiliations:** Department of Cardiopneumology, Heart Institute, School of medicine, University of São Paulo, São Paulo, Brazil

## Abstract

**Introduction:**

Cognitive dysfunction with heart failure with reduced ejection fraction (HFrEF) is well studied. However, there are few comparative studies with heart failure and preserved ejection fraction (HFpEF). Cognitive dysfunction diagnosis usually demands a long neuropsychological battery. We developed MentalPlus® digital game to overwhelm that issue.

**Methods:**

As a pilot study, we evaluated 60 patients with systemic hypertension and HFpEF. They were submitted to TICS (Telephone Interview Cognitive Status) to evaluate the general cognitive function and 25 minutes of MentalPlus® digital game evaluation.

**Results:**

The results disclosed 60 hypertensive patients. All of them presented with HFpEF. Patients presented a mean age of 56±10 years; 46% male; LVMi (g/m^2^) mean of 110±20; educational attainment of 9 years or more; mean income of 8 Brazilian minimum wages. The TICS results disclosed 28 ±3.7. MentalPlus® digital game evaluation disclosed preserved values for the phases I, III, IV, V, VI, and VII. Phase II, short-term memory related, was below the normals values that were assigned. This group of patients presented a normal general cognition by both evaluations, except for specific functions displayed above, disclosed by MentalPlus®. The MentalPlus® was designed to possibly evaluate specific cognitive functions separately, like attention, memory, executive function, and language, because each phase evaluates specific functions shortly.

**Conclusion:**

Hypertensive HFpEF patients presented in general a normal cognition, except for some aspects related to short-term memory. The MentalPlus® digital game, compared with TICS, presented similar general results. It is an advantage that MentalPlus® software could be used to assess cognitive function, in general and individually, and be an open tool shortly.

## 1. Introduction

Heart failure (HF) is a major health problem in developed countries that affects 1%–2% of the adult population with rising prevalence in people over 65 years or older [1].

There is a strong association with increased mortality and morbidity, frequent hospital admissions, and reduced quality of life and functional status [2]. It is important to bear in mind that most randomized controlled trials in HF specifically exclude patients with significant comorbidities or HFpEF, yet this group constitutes a significant proportion of older people with HF [3–5].

Up to 75 % of >65-year-old patients will have multiple chronic conditions that will impact HF management; [4, 6] comorbidity is one of the strongest independent predictors of rehospitalization and mortality[7]. One of the key factors less frequently considered as comorbidities in patients with HF is cognitive impairment.

The impaired cognitive function is associated with poor levels of self-care and functional decline [8] and predicts nonengagement in HF management programmers. Patients may not recognize or understand a change in symptoms or function, with negative consequences for overall care.

Identifying cognitive impairment allows patient, caregivers, and clinicians to plan management strategies and clinicians should be vigilant for its presence. Observational data suggest that informal assessment of cognition by cardiologists is insensitive, with approximately three in four HF patients with important cognitive problems not recognized during their routine consultations. [9] Nearly half of all patients with HF symptoms, like breathlessness on exertion, have a preserved EF, exceeding 50%. Its prevalence is rising, with morbidity, mortality, and healthcare costs following the same trends of HF with reduced EF (HFrEF). HFpEF patients also present multiple common comorbidities, such as hypertension, diabetes mellitus, renal disease, atrial fibrillation, and metabolic syndrome, which have a major impact on the syndrome and mortality. [6, 10–13]

Cognitive function is a superior cortical function involving multiple brain activities that permit that human being perceives information, learn it, and remember specific knowledge. It can enable us to solve problems and plan actions during daily life. There are some cognitive functions, including memory, attention, executive function, language, and visuospatial ability. [14]The most frequently measured cognitive domains in HF studies are attention, working memory, long-term memory, learning, executive function, and psychomotor speed, with approximately 28%–58% of individuals with HF demonstrating impairment of one or more of those cognitive domains. [15] There are many neuropsychologic tests, used to measure different cognitive domains. The main impairment is that it is a time-consuming process. It usually deserves around 2 hours to have a complete evaluation, and only specialists can make it. Moreover, at least in our country, Brazil, the majority of these tests can be known and applied only by psychologists and neuropsychologists. Other health professionals interested in evaluating the cognitive function of patients are not allowed to use them. The restriction to use and time-consuming applicability of the neuropsychological tests has led the scientific community to a constant search for alternative diagnostic methods to evaluate cognition. As an alternative to overcome those issues, we developed a digital game named MentalPlus® as a possible open tool to assess cognition. Previous studies have shown that digital games might stimulate cognitive functions and enhance skills such as creativity, the search for strategies, decision-making, and skills aimed at visuoperception. In clinical trials, the games have been applied for neuropsychological function skill improvement. They have been able to modify the structure and functioning of the brain architecture. [16, 17]

However, the use of virtual games for assessing the integrity of neuropsychological functions is insufficient or nonexistent. The development and validation of an easy neuropsychological open test application as a digital game can contribute to a better cognition knowledge in different clinical situations, as in HFpEF.

The primary objective of this study aimed to evaluate general cognitive status comparing the MentalPlus® digital game to Telephone Interview Cognition Status (TICS). Afterward, the secondary objective of the study would be to describe specific cognitive function status in this pilot study group and stimulate the continuous study to validate that simple, open source tool to be used by colleagues in various health fields to access cognitive dysfunction in earlier states. One of the most important social conditions would be its application mainly where the sociodemographic conditions are low and give a chance to those patients to have rehabilitation and possible inclusion in society.

## 2. Materials and Methods

### 2.1. Study Design and Subject Enrollment

The study conceived as a pilot exploratory study, received the approval of the Ethics Committee for Research Project Analysis (CAPPesq) of the Clinical Board, Hospital das Clínicas da Faculdade de Medicina da Universidade de São Paulo (HC-FMUSP) (S1 File).

There were 60 hypertensive treated patients, presenting exertion dyspnea, enrolled to be submitted to TICS, and play a free digital game, MentalPlus®, after invitation acceptance and a signed written informed consent. The volunteer recruitment to the study was from May 2016 to August 2016. The neuropsychological assessment phase study finished in September 2016.

**Figure 1:**
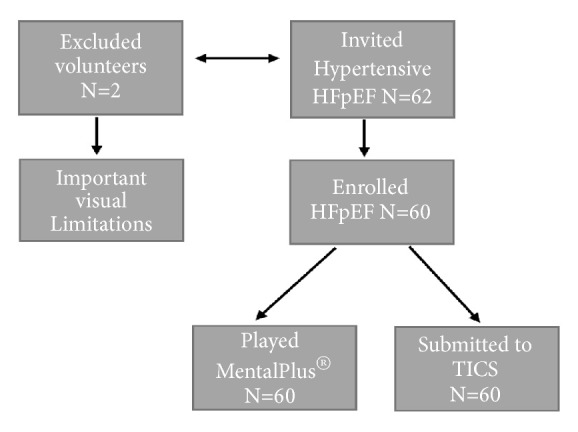
**Study fluxogram. **HFpEF: heart failure with preserved ejection fraction; MentalPlus®: Open Tool Virtual Game; TICS: Telephone Interview Cognition Status.

Inclusion criteria were hypertensive treated patients who accepted the invitation to play a free digital game, MentalPlus®, and receive TICS test. They should present dyspnea on exertion and a normal left ventricular systolic function (Figure 1).

60 transthoracic echocardiography examinations performed with a 2D, M-Mode, pulse wave Doppler, and tissue Doppler echocardiography by using an ACUSON X300™ ultrasound system, Mountain View (California, USA) with a 1.4–5.6 MHz frequency bandwidth transducer. The echocardiographic measurements were according to American Society of Echocardiography: guidelines: http://asecho.org/guidelines/guidelines-standards/. Myocardial velocity profiles of the lateral mitral annulus are obtained by placing a 6 mm sample volume at the junction of the mitral annulus and lateral myocardial wall. Left ventricular mass (LVM) was calculated using the Devereux Formula [18], where LVM = 0.8(1.04(IVST + LVID + LPWT)^3^ – (LVID)^3^ + 0.6), LVST is interventricular septal thickness, LVID is left ventricular internal dimension, and LPWT is left posterior wall thickness. The left ventricular mass index (LVMI) calculus used the formula, LVM/(height). [18, 19] The systolic ventricular function was assessed by 2D biplane Simpson's rule. Left ventricular ejection fraction (LVEF)** ≥ **50 % has been defined as preserved or normal left ventricular (LV) systolic function.

The exclusion criteria were the presence of a history of brain disease or dementia, psychiatric disorders that affect cognition, and the presence of some grade of visuomotor ability impairment to play video games and a not preserved left ventricular systolic function.

The Telephone Interview Cognition Status (TICS) was applied to all patients before MentalPlus®. The test consists of an interview script with 11 items that assess spatial and temporal orientation skills, mind control, memory, general information, language, and calculations (S2 File). The TICS total score is a sum of the individual item scores. It provides a measure of global cognitive functioning and can be used to monitor changes in cognitive functioning over time. TICS results are reported using a qualitative impairment range and* T* scores (TICS normal range for Brazilian population 27.7±3.3). The appropriate normative reference group for interpretation will depend on the reason for the evaluation and the examinee's age and level of education [20–22]. The study followed the CONSORT criteria (http://www.consort-statement.org/) (S3 File).

### 2.2. The Video Game MentalPlus® Characteristics

MentalPlus® is a digital game developed to evaluate and stimulate neuropsychological functions, patented and registered with the National Library Foundation by Law no. 9,610/98, under copyright no. 663,707. This digital game identifies the neuropsychological deficits in functions, Attention/Memory/Executive. Attention, resistance to distractor stimulus, sustaining attention, and memory evaluation consist of a series of tasks in 8 digital game phases. We show the game phases in detail in the video (S4 File).

### 2.3. Sociodemographic Evaluation

Demographic data were analyzed, including age, education, and family income. The education evaluation was an index derived from the schooling years and the family income based on Brazilian minimum wages.

## 3. Statistical Analyses

The qualitative characteristics were described using absolute and relative frequencies and the quantitative characteristics were described using summary measures (mean, standard deviation, median, minimum, and maximum) (Kirkwood and Sterne, 2006).

The domains of cognition assessed by MentalPlus were described according to each assessed personal and clinical qualitative trait and compared among the categories using Mann-Whitney tests (Kirkwood and Sterne, 2006) to verify construct validation of MentalPlus.

The Spearman (Kirkwood and Sterne, 2006) correlations of the MentalPlus domains with quantitative characteristics, age, and MAP (presence of HFpEF and presence of diastolic dysfunction) were also calculated for construct validation as well as for TICs for the concurrent validation of the MentalPlus domains with another cognition scale. But as that study was a descriptive and exploratory one, the group had still not the pretension to evaluate the power of the test at that study stage. We have the intention of developing larger studies in a newer future. At that time, surely, a precise sample size as confidence intervals and power calculations will be executed for more precise results. With that pilot study, the main objective was to show some data with hypertensive patients with HFpEF that could stimulate the scientific community to study cognitive function deeply in many heart groups, possibly with the open tool MentalPlus® digital game.

The analyses were performed using IBM-SPSS for Windows software version 20.0 and tabulated using Microsoft-Excel 2003 software and tests were performed with a significance level of 5% [23].

## 4. Results

According to Figure 1, we recruited 60 hypertensive patients under treatment with HFpEF and excluded two patients due to limiting visual problems. Patients presented treated hypertension and dyspnea on exertion. There was no history of anemia or COPD.  Table 1 discloses sociodemographic and clinical data, with the mean and standard deviation (SD). The 60 study participants presented a mean age of 56±10 years (mean; SD). 46% were male. They had a school level mean of 13±3 years (mean; SD) and 72 % received a mean income ≥ ten while 28% received an amount corresponding to ≥ 5 and < 10 Brazilian minimum wages per month. The drugs used were thiazide diuretics, calcium channel blockers, beta-blockers, angiotensin-converting enzyme (ACE) inhibitors, and angiotensin II receptor blockers (ARB).  Table 2 presented some clinical and echocardiographic parameters. Mean medium arterial pressure (MAP) was 94±9mmHg (mean; SD), with antihypertensive medication. They all presented EF ≥ 50% by 2D biplane Simpson's rule and 100% disclosed an abnormal diastolic function by tissue Doppler velocity measurements. Left ventricular hypertrophy was present in 53%, with a left ventricular mass index (LVMi) >90 and 110 g/m^2^ for women and men, respectively. Left atrium volume per square meter was larger than 30 ml in 53% of them. Different professionals supervised both tests for each patient. We have not evaluated NT-proBNP.

In the neuropsychological evaluation, the HFpEF group exhibited TICS score of 28±3 (mean; SD), for an expectation of more than 25±3 (mean; SD) for the normal population [23].

Table 3 displayed that there was no statistically significant correlation between the MentalPlus domains and the age of the hypertensive patients (p> 0.05) and only the inverse correlation between short-term memory, MentalPlus® Phase II with mean arterial pressure (MAP) (r = -0.290 and p = 0.025). That implies that the higher the MAP, the lower the values for short-term memory. Although the correlation was low, it was statistically significant. TICs showed no statistically significant correlation with any of the MentalPlus domains (p> 0.05). We tried to show that patients with hypertension and HFpEF with normal general cognitive function, evaluated by TICS, could have specific deficiencies as showed by MentalPlus® phase, like short memory function.

## 5. Discussion

This study found evidence that we might use MentalPlus® digital game in an HFpEF group of patients, for neuropsychological evaluation. It can be an open diagnostic tool of cognitive dysfunction for mnemonic, attentional, and executive functions. Data analysis yielded interesting findings. There was a good agreement for selective and alternate attention, long-term memory, inhibitory control, executive function, and visuoperception and visuoconstruction. Only short-term memory displayed an inverse correlation with MAP. Although the correlation was low, it was statistically significant (Table 3).

The use of the game in patients with HFpEF disclosed a normal general cognition, except for some aspects of the memory. That might be a sign that even in patients with normal ventricular systolic function, some factors that might be more intensively evaluated by MentalPlus® digital game can disclose characteristics that TICS by itself is not able to identify. The main interest in developing that possible open tool for cognitive function evaluation is its convenience when compared with extensive and time-consuming psychological battery usually undertaken to that end.

The digital game presents many advantages through the extensive psychological battery usually performed to evaluate those patients. It only requires about 25 minutes to be played through all its phases (S4). It would be enough to have an oriented health professional to apply the video game. The MentalPlus® has the proposal to evaluate attention, short- and long-term memory, executive function, language, and visuoconstruction. We can have both general and individualized evaluation of cognitive function. It can be used many times during cognitive evaluation follow-up and follow small changes in cognitive functions separately. Other usual cognitive tests, like Mini-Mental State Examination (MMSE), could be speculated to be applied. Otherwise, the content of the MMSE is highly verbal, lacking sufficient items to measure both visuospatial and constructional praxis adequately. It brings some limitations as evaluation of low cognitive impairment as small changes. Hence, its utility to detect impairment caused by focal lesions is uncertain. We are aware that to evaluate all those cognitive functions, it would be necessary a long time to perform a neuropsychological battery.

Therefore, that justification would favor MentalPlus® digital game usefulness in a broad of clinical settings as we can observe in this pilot study with an emphasis in HFpEF, enabling a prompt cognitive dysfunction diagnosis. Future research might focus on direct neuropsychological assessment using the MentalPlus® digital game in patients for diverse underline pathologies. We already presented the game reliability in near past [23]. Now, our next step is the validation of the digital game cited in a normal population, compared with a normal neuropsychologic test battery. Although preliminary studies have shown that we can apply that digital game for that goal, there is a necessity for accurate measurements and calibrations to get reliable results of these cognitive functions.

## 6. Conclusion

The MentalPlus® digital game already presented reliable evidence for cognition evaluation like attention, memory, and executive functions. Here we showed that in HFpEF the general and specific cognitive functions are preserved, except for a lower range of short-term memory function-MentalPlus phase II. It might have many implications but bring to the scientific community that at that level of the pathology we should make every effort to stimulate the cognitive functions, besides the maintenance of the treatment and all the risk factors. As a feasible open tool, it is possible to claim for new colleagues to use the digital game and evaluate it with us. Additional study will contribute for an effective calibration of the game as for its validation and usual open utilization.

## Figures and Tables

**Table 1:** Sociodemographic and clinical data.

Age(years) (Mean; SD)	56±10
Male Gender (%)	46

School level in years (13±3) (Mean; SD) (%)	100%

Mean Income ≥10 minimum wage (%)	75%

Dyspnea on exertion (%)	100%

Presence of anemia (%)	0%

Presence of POCD (%)	0%

Past history of heart failure (%)	0%

Presence of Type II Diabetes (%)	25%

Presence of 26≤ BMI ≤ 33	00%

Presence of Hypertension (%)	00%

Use of thiazide diuretics, calcium channel blockers, ARB, ACE inhibitors, beta-blockers (alone or in combination)	100%

Number of evaluated patients	60

SD: standard deviation; BMI: Body Mass Index; ARB: angiotensin receptor blocker; ACE: angiotensin converting enzyme inhibitors.

**Table 2:** Parameters of echocardiography evaluation.

MAP (mmHg) (Mean; SD)	VMI>90g/m^2^ for female or >115g/m^2^ for male (percentage)	LVEF (Simpson)≥50% (percentage)	LA volume (ml/m^2^)≥30 (percentage)	E/A filling pressure>1 (percentage)	E/e ′≥13 or E/e ′≤8 (percentage)
(94;9)	53%	100%	53%	47%	100%

MAP: mean arterial pressure; SD: standard deviation; VMI: ventricular mass index; LVEF: left ventricular ejection fraction; Simpson: Simpson's method; LA: left atrium.

**Table 3:** Spearman correlation with MentalPlus® phases, with patients age, mean arterial pressure, and TICS.

**Spearman Correlation**		Age (years)	Mean arterial pressure	TICS
Short Term Memory, MentalPlus Phase II	r	−0,048	−0,290	0,119
	p	0,715	**0,025**	0,363
Inhibitory Control, MentalPlus Phase II	r	0,092	0,033	0,097
	p	0,485	0,801	0,463
Visuoperception and Visuoconstruction, MP phases VI and VII	r	0,057	0,016	−0,151
	p	0,663	0,904	0,248
Selective Attention, MP phases III and IV	r	0,093	−0,044	0,097
	p	0,480	0,741	0,460
Alternate Attention, MP phases V and VI	r	−0,009	0,054	0,192
	p	0,944	0,680	0,141
Long Phase Memory, MP phase I	r	−0,182	0,033	0,122
	p	0,163	0,804	0,352

Spearman correlation				

MP: Mentalplus.

## Supplementary Materials

Supplementary Materials Supporting File 1 (S1): the approval of the Ethics Committee for Research Project Analysis (CAPPesq) of the Clinical Board, Hospital das Clínicas da Faculdade de Medicina da Universidade de São Paulo (HC-FMUSP). Supporting File 2 (S2): the Telephone Interview Cognition Status (TICS). Supporting File 3 (S3): the CONSORT criteria (http://www.consort-statement.org). Supporting File 4 (S4): video-demo MentalPlus®.

## References

[1] Ponikowski P., Voors A. A., Anker S. D. (2016). 2016 ESC guidelines for the diagnosis and treatment of acute and chronic heart failure: The task force for the diagnosis and treatment of acute and chronic heart failure of the European Society of Cardiology (ESC) developed with the special contribution of the Heart Failure Association (HFA) of the ESC. *European Heart Journal*.

[2] Maggioni A. P., Dahlström U., Filippatos G. (2014). EURObservational Research Programme: regional differences and 1-year follow-up results of the Heart Failure Pilot Survey (ESC-HF Pilot). *European Journal of Heart Failure*.

[3] Donkor A., Cleland J., McDonagh T. National Heart Failure Audit, April 2014–March 2015. http://www.ucl.ac.uk/nicor/audits/heartfailure/documents/annualreports/heartfailurepublication14_15.

[4] Sharma K., Kass D. A. (2014). Heart failure with preserved ejection fraction: mechanisms, clinical features, and therapies. *Circulation Research*.

[5] Yancy C. W., Jessup M., Bozkurt B. (2013). 2013 ACCF/AHA guideline for the management of heart failure: A report of the American college of cardiology foundation/American heart association task force on practice guidelines. *Circulation*.

[6] Go A. S., Mozaffarian D., Roger V. L. (2013). American Heart Association Statistics Committee and Stroke Statistics Subcommittee. Heart disease and stroke statistics–2013 update: a report from the American Heart Association. *Circulation*.

[7] Oudejans I., Mosterd A., Zuithoff N. P., Hoes A. W. (2012). Comorbidity drives mortality in newly diagnosed HF: a study among geriatric outpatients. *Journal of Cardiac Failure*.

[8] Harkness K., Heckman G. A., Akhtar-Danesh N., Demers C., Gunn E., McKelvie R. S. (2014). Cognitive function and self-care management in older patients with heart failure. *European Journal of Cardiovascular Nursing*.

[9] Hanon O., Vidal J.-S., de Groote P. (2014). Prevalence of memory disorders in ambulatory patients aged ≥70 years with chronic heart failure (from the EFICARE Study). *The American Journal of Cardiology*.

[10] Steinberg B. A., Zhao X., Heidenreich P. A. (2012). Trends in patients hospitalized with heart failure and preserved left ventricular ejection fraction: Prevalence, therapies, and outcomes. *Circulation*.

[11] Owan T. E., Hodge D. O., Herges R. M., Jacobsen S. J., Roger V. L., Redfield M. M. (2006). Trends in prevalence and outcome of heart failure with preserved ejection fraction. *The New England Journal of Medicine*.

[12] Bhatia R. S., Tu J. V., Lee D. S. (2006). Outcome of heart failure with preserved ejection fraction in a population-based study. *The New England Journal of Medicine*.

[13] Liao L., Jollis J. G., Anstrom K. J. (2006). Costs for heart failure with normal vs reduced ejection fraction. *Arch Intern Med*.

[14] Lezak M. D. (2004). *Neuropsychological assessment*.

[15] Bauer L., Pozehl B., Hertzog M., Johnson J., Zimmerman L., Filipi M. (2017). A brief neuropsychological battery for use in the chronic heart failure population. *European Journal of Cardiovascular Nursing*.

[16] Green C., Bavelier D. (2012). Learning, Attentional Control, and Action Video Games. *Current Biology*.

[17] Fernández-Aranda F., Jiménez-Murcia S., Santamaría J. J. (2012). Video games as a complementary therapy tool in mental disorders: playMancer, a European multicentre study. *Journal of Mental Health*.

[18] Devereux R. B., Alonso D. R., Lutas E. M. (1986). Echocardiographic assessment of left ventricular hypertrophy: comparison to necropsy findings. *American Journal of Cardiology*.

[19] Feldstein C. A., Akopian M., Olivieri A. O., Kramer A. P., Nasi M., Garrido D. (2005). A comparison of body mass index and waist-to-hip ratio as indicators of hypertension risk in an urban Argentine population: A hospital-based study. *Nutrition, Metabolism & Cardiovascular Diseases*.

[20] Fong T. G., Fearing M. A., Jones R. N. (2009). Telephone Interview for Cognitive Status: Creating a crosswalk with the Mini-Mental State Examination. *Alzheimer’s & Dementia*.

[21] Lopez O. L., Kuller L. H. (2010). Telephone interview for cognitive status. *Neuroepidemiology*.

[22] Brandt J., Spencer M., Folstein M. (1988). The Telephone Interview for Cognitive Status. *Neuropsychiatry Neuropsychol Behav Neurol*.

[23] kirkwood B. R., sterne J. A. C. (2006). *Essential medical statistics*.

