# Supplementary material for: The MentalPlus® Digital Game Might Be an Accessible Open Source Tool to Evaluate Cognitive Dysfunction in Heart Failure with Preserved Ejection Fraction in Hypertensive Patients: A Pilot Exploratory Study
Source: Int J Hypertens. 2018 Aug 6;2018:6028534. doi: 10.1155/2018/6028534 (PMC6106805; doi:10.1155/2018/6028534)
Supplement: Supplementary Materials — Supporting File 1 (S1): the approval of the Ethics Committee for Research Project Analysis (CAPPesq) of the Clinical Board, Hospital das Clínicas da Faculdade de Medicina da Universidade de São Paulo (HC-FMUSP). Supporting File 2 (S2): the Telephone Interview Cognition Status (TICS). Supporting File 3 (S3): the CONSORT criteria (http://www.consort-statement.org). Supporting File 4 (S4): video-demo MentalPlus®. [file 6028534.f1.zip › S1 Comissaoetica.pdf]

PARECER CONSUBSTANCIADO DO CEP

DADOS DO PROJETO DE PESQUISA

Título da Pesquisa: Jogo digital MentalPlus® para avaliação da função cognitiva perioperatória: análise de conteúdo, consistência interna, validade convergente e validação clínica do teste diagnóstico

Pesquisador: Lívia Stocco Sanches Valentin

Área Temática:

Versão: 2

CAAE: 49463315.5.1001.0068

Instituição Proponente: HOSPITAL DAS CLINICAS DA FACULDADE DE MEDICINA DA U S P

Patrocinador Principal: HOSPITAL DAS CLINICAS DA FACULDADE DE MEDICINA DA U S P
FUNDACAO DE AMPARO A PESQUISA DO ESTADO DE SAO PAULO

DADOS DO PARECER

Número do Parecer: 1.482.094

Apresentação do Projeto:

A disfunção cognitiva é evento adverso frequente no período pós-operatório, especialmente em pacientes idosos. Os testes neuropsicológicos habitualmente utilizados para o diagnóstico de disfunção cognitiva pós-operatória são de aplicação demorada e com sensibilidade e especificidade variáveis, dificultando o uso rotineiro. A dificuldade de aplicação da bateria de testes cognitivos pré-operatórios diminui a viabilidade da adoção de medidas para aumento da reserva cognitiva pré-operatória e de métodos para prevenção, diagnóstico e reabilitação dos casos de POCD, sendo desejável a procura por métodos diagnósticos alternativos. Os jogos digitais tem potencial efeito neuromodulador e tem sido utilizados como alternativa para o tratamento psicoterapêutico e para a reabilitação das habilidades cognitivas. Entretanto, o uso destes jogos para avaliação da integridade das funções neuropsicológicas perioperatórias ainda é inexistente. Hipotetizou-se que um jogo digital especificamente criado e de fácil aplicação prática possa ser utilizado como teste neuropsicológico para o diagnóstico da disfunção cognitiva pós-operatória. OBJETIVO: O objetivo deste estudo é a validação de um jogo digital específico (MentalPlus®) para avaliação cognitiva e avaliação de seu uso para o diagnóstico
de disfunção cognitiva pós-operatória. MÉTODOS Serão estudados sujeitos maiores de 20 anos,

Endereço: Rua Ovídio Pires de Campos, 225 5º andar
Bairro: Cerqueira Cesar **CEP:** 05.403-010
UF: SP **Município:** SAO PAULO
Telefone: (11)2661-7585 **Fax:** (11)2661-7585 **E-mail:** cappesq.adm@hc.fm.usp.br

alfabetizados, de ambos os gêneros e candidatos à cirurgia sob anestesia geral no Hospital das Clínicas da Faculdade de Medicina da Universidade de São Paulo. Serão controladas as variáveis idade, gênero e escolaridade. Os voluntários serão submetidos à aplicação de testes para a memória de curto e longo prazo (VLT), memória visual (TEM-R), para as funções executivas (SCWST) e para a atenção seletiva e alternada (TMT-A/B), BETA II para avaliação do cognitivo global, WOQOL para Qualidade de Vida e BDI-II para avaliação dos sintomas depressivos, além do jogo digital MentalPlus®. Será utilizada a ressonância magnética funcional (fMRI) para avaliar a associação entre a resposta cerebral e o treinamento cognitivo pelo jogo digital MentalPlus®. Serão realizadas análises de confiabilidade e de validade do jogo digital com o intuito de suprir a carência no âmbito da avaliação de funções cognitivas de maneira lúdica e prática e disponibilizar a avaliação neuropsicológica à profissionais da área da saúde, médicos anestesiologistas e cirurgiões.

Objetivo da Pesquisa:

Objetivo Primário: Validar o jogo digital MentalPlus® como teste neuropsicológico para a avaliação cognitiva pré e pós-operatórias.Esta validação será feita comparando as fases do MentalPlus® em seus resultados com os dados dos testes neuropsicológicos já validados para as funções –executiva; memória e atenção. Avaliando o uso deste recurso diagnóstico na avaliação cognitiva de pacientes candidatos a procedimentos cirúrgicos, comparando-o com testes já padronizados e validados para avaliar as funções executivas, mnemônicas e atencionais.Objetivo Secundário: • Estudar a influência da idade, do gênero e do grau de escolaridade de pacientes cirúrgicos no desempenho para o uso de aparelhos eletrônicos
(notebook, tablets, celulares) e jogos digitais.• Estudar a consistência interna e as validades de conteúdo e de constructo (convergente) do MentalPlus® nos pacientes avaliados.Como um estudo interventivo em pacientes no pós-operatório:• Investigar a capacidade de reabilitação das funções cognitivas após sessões de treino com o jogo MentalPlus®. •Avaliar o efeito do treino com jogo MentalPlus® em redes cerebrais envolvidas com função executiva, memória e atenção por meio de fMRI.

Avaliação dos Riscos e Benefícios:

Riscos: Durante a condução do estudo não esperamos riscos aos pacientes além daqueles previstos pelo procedimento anestésico cirúrgico. Porém pode ser que durante a bateria dos testes neuropsicológicos os pcts apresentem cansaço, mas faremos várias pausas e retomaremos os testes apenas quando o paciente se sentir confortável para responder as questões e jogar o game digital.Benefícios: O benefício deste estudo é encontrar outra alternativa mais funcional de

diagnóstico das principais disfunções cognitivas no perioperatório de cirurgia não cardíaca. Mas apenas ao final do estudo poderemos comprovar nossa hipótese.

Comentários e Considerações sobre a Pesquisa:

Todos os questionamentos do parecer anterior foram atendidos pela pesquisadora.

Considerações sobre os Termos de apresentação obrigatória:

Adequado.

Recomendações:

Conclusões ou Pendências e Lista de Inadequações:

Sem pendências.

Considerações Finais a critério do CEP:

Em conformidade com a Resolução CNS nº 466/12 – cabe ao pesquisador: a) desenvolver o projeto conforme delineado; b) elaborar e apresentar relatórios parciais e final; c)apresentar dados solicitados pelo CEP, a qualquer momento; d) manter em arquivo sob sua guarda, por 5 anos da pesquisa, contendo fichas individuais e todos os demais documentos recomendados pelo CEP; e) encaminhar os resultados para publicação, com os devidos créditos aos pesquisadores associados e ao pessoal técnico participante do projeto; f) justificar perante ao CEP interrupção do projeto ou a não publicação dos resultados.

Este parecer foi elaborado baseado nos documentos abaixo relacionados:

Tipo Documento	Arquivo	Postagem	Autor	Situação
Informações Básicas do Projeto	PB_INFORMAÇÕES_BÁSICAS_DO_PROJETO_543755.pdf	22/02/2016 14:44:20		Aceito
Folha de Rosto	folha_De_Rosto_Mental_Pus_Livia_signed.pdf	22/02/2016 14:41:14	Lívia Stocco Sanches Valentin	Aceito
Declaração de Pesquisadores	Ehab_Letter.pdf	16/02/2016 23:57:31	Lívia Stocco Sanches Valentin	Aceito
Declaração de Pesquisadores	Eriksson_Letter.pdf	16/02/2016 23:19:31	Lívia Stocco Sanches Valentin	Aceito
Declaração de Pesquisadores	Dr_Andre_Brunoni.pdf	16/02/2016 23:02:24	Lívia Stocco Sanches Valentin	Aceito
Declaração de Pesquisadores	Letter_Fregni.pdf	16/02/2016 23:00:29	Lívia Stocco Sanches Valentin	Aceito
Declaração de Pesquisadores	Fernando_Gomes_Pinto_IPq.pdf	16/02/2016 22:57:35	Lívia Stocco Sanches Valentin	Aceito
Declaração de Pesquisadores	Lars_Simon_Rasmussen_Letter.pdf	16/02/2016 22:54:21	Lívia Stocco Sanches Valentin	Aceito

Declaração de Pesquisadores	Kuhn_letter.pdf	16/02/2016 22:52:03	Lívia Stocco Sanches Valentin	Aceito
Declaração de Pesquisadores	Letter_Kalkman.pdf	16/02/2016 22:50:18	Lívia Stocco Sanches Valentin	Aceito
Declaração de Pesquisadores	Inclusao_de_Centros_de_Pesquisa_MENTALPLUS.pdf	14/02/2016 15:02:36	Lívia Stocco Sanches Valentin	Aceito
Declaração de Pesquisadores	Siamak_Rahman_mentalplus_letter.pdf	14/02/2016 13:39:25	Lívia Stocco Sanches Valentin	Aceito
Outros	Memo_respostas_parecer_CAPpesq_MentalPlus.pdf	14/02/2016 13:18:27	Lívia Stocco Sanches Valentin	Aceito
Outros	Cadastro_online_assinado_pelo_departamento.pdf	09/09/2015 13:06:25	Suely Pereira Zeferino	Aceito
Outros	Lista_de_centros_participantes_estude_Mental_Plus_3_signed.pdf	04/09/2015 09:43:06	Suely Pereira Zeferino	Aceito
Projeto Detalhado / Brochura Investigador	MetalPlus versãoo 4.0 de 10.08.15.pdf	17/08/2015 08:26:15		Aceito
Outros	Termo_anuencia_Centro_participante_InRad_rev_eds-5.pdf	14/08/2015 12:45:32		Aceito
TCLE / Termos de Assentimento / Justificativa de Ausência	TCLE- Mental Plus Versão 1.0 (03.05.15).docx	14/08/2015 12:37:32		Aceito

Situação do Parecer:
Aprovado

Necessita Apreciação da CONEP:
Não

SAO PAULO, 07 de Abril de 2016

Assinado por:
ALFREDO JOSE MANSUR
(Coordenador)

Endereço: Rua Ovídio Pires de Campos, 225 5º andar
Bairro: Cerqueira Cesar **CEP:** 05.403-010
UF: SP **Município:** SAO PAULO
Telefone: (11)2661-7585 **Fax:** (11)2661-7585 **E-mail:** cappesq.adm@hc.fm.usp.br
