# Supplementary material for: The MentalPlus® Digital Game Might Be an Accessible Open Source Tool to Evaluate Cognitive Dysfunction in Heart Failure with Preserved Ejection Fraction in Hypertensive Patients: A Pilot Exploratory Study
Source: Int J Hypertens. 2018 Aug 6;2018:6028534. doi: 10.1155/2018/6028534 (PMC6106805; doi:10.1155/2018/6028534)
Supplement: Supplementary Materials — Supporting File 1 (S1): the approval of the Ethics Committee for Research Project Analysis (CAPPesq) of the Clinical Board, Hospital das Clínicas da Faculdade de Medicina da Universidade de São Paulo (HC-FMUSP). Supporting File 2 (S2): the Telephone Interview Cognition Status (TICS). Supporting File 3 (S3): the CONSORT criteria (http://www.consort-statement.org). Supporting File 4 (S4): video-demo MentalPlus®. [file 6028534.f1.zip › S2fleTICS.pdf]

Mental Plus

Mental Plus

Name: ____

ID:______________________Phone:_____________________________________

Evaluator: ____

Date: _____/_______/_______ Phase: □Pre □Pos □7th day □30th day □90th day

Evaluation □ **Rehabilitation □**

TICS- Telephone Interview Cognitive Status

(1 point for each **correct** answer / **0** for **wrong** answers)

1. ORIENTATION

A. What day of the month is it? __; What day of the week is today? __ (__/2)

B. Which today's date? (day, month and year) _____ / _______ / ________ (___ / 3)

C. In which season are we? (__/1)

□ Spring □ Summer □Autumn □Winter (____/4)

D. How old are you? _____years. (__/1)

E. What's your phone number? ____________________________ (__/1)

2. IMMEDIATE MEMORY

F. I will read a list of 10 words for you. Please listen carefully to the 10 words. Then I want you to talk to me all the words that you remember, right? (___ / 10)

□ cabin	□ pipe	□ elephant	□ chest	□ silk
□ theatre	□ watch	□ whip	□ giant	□ pillow

G. Now, without using pencil and paper to do some math just thinking, okay?
100-7 is? ____. 93-7 is? _____.

Now continue decreasing the number 7 is left until I ask them to stop...
___ (86) -7 ____ (79) -7 ____ (72) -7 ____ (65)-7 (58) (___ / 7)

NOTE: If the volunteer missing the previous number but hit the next subtractions consider a mistake and the next hit. Eg: 100-7 = 73 If the patient provide the number 74 but the next subtraction hit the result, consider a hit. 74-7 = 67 (1 point)

H. Please count from 1 to 20, but backwards.

20, 19, 18, 17, 16, 15, 14, 13, 12, 11, 10, 09, 08, 07, 06, 05, 04, 03, 02, 01. (___ / 1)

3. EPISODIC MEMORY

I. What people often use to cut paper? (Scissors) (___ / 1)
J. What is the name of the plant full thorn found in the desert? (Cacti) (__ / 1)
K. Who is the current president of ______________________? _____________ (___/1)
L. Who is the current vice president of _______________________________? (___/1)
If the volunteer does not remember, ask who is the current governor of the state.
N. What is the antonym or otherwise (opposite) of Eastern? West (___ / 1)

4. LANGUAGE FOR REPLAY

O. Please listen and then repeat:

Methodist Episcopal (___ / 1)

5. MEMORY EVOCATION (LATE)

P. Try to remember and tell me all the words of list I talked about earlier.

□ cabin	□ pipe	□ elephant	□ chest	□ silk
□ theater	□ watch	□ whip	□ giant	□ pillow (___ / 10)

TOTAL: ___ / 40
