# Supplementary material for: The MentalPlus® Digital Game Might Be an Accessible Open Source Tool to Evaluate Cognitive Dysfunction in Heart Failure with Preserved Ejection Fraction in Hypertensive Patients: A Pilot Exploratory Study
Source: Int J Hypertens. 2018 Aug 6;2018:6028534. doi: 10.1155/2018/6028534 (PMC6106805; doi:10.1155/2018/6028534)
Supplement: Supplementary Materials — Supporting File 1 (S1): the approval of the Ethics Committee for Research Project Analysis (CAPPesq) of the Clinical Board, Hospital das Clínicas da Faculdade de Medicina da Universidade de São Paulo (HC-FMUSP). Supporting File 2 (S2): the Telephone Interview Cognition Status (TICS). Supporting File 3 (S3): the CONSORT criteria (http://www.consort-statement.org). Supporting File 4 (S4): video-demo MentalPlus®. [file 6028534.f1.zip › SF4Fig.8.supportingFile4.docx]

**Supporting file 4 (S4): Video-demo MentalPlus®**
